# Dual Inhibition of Autophagy and PI3K/AKT/MTOR Pathway as a Therapeutic Strategy in Head and Neck Squamous Cell Carcinoma

**DOI:** 10.3390/cancers12092371

**Published:** 2020-08-21

**Authors:** Monique Bernard, Guillaume B. Cardin, Maxime Cahuzac, Tareck Ayad, Eric Bissada, Louis Guertin, Houda Bahig, Phuc Felix Nguyen-Tan, Edith Filion, Olivier Ballivy, Denis Soulieres, Francis Rodier, Apostolos Christopoulos

**Affiliations:** 1 Centre de Recherche du Centre Hospitalier de l’Université de Montréal (CRCHUM), Montreal, QC H2X 0A9, Canada; monique.bernard.chum@ssss.gouv.qc.ca (M.B.); cardinguill@gmail.com (G.B.C.); maxime.cahuzac@umontreal.ca (M.C.); ayad_t@hotmail.com (T.A.); houda.bahig.chum@ssss.gouv.qc.ca (H.B.); rodierf@mac.com (F.R.); 2 Institut du Cancer de Montréal (ICM), Montreal, QC H2X 0A9, Canada; 3 Otolaryngology-Head and Neck Surgery Service, Centre Hospitalier de l’Université de Montréal (CHUM), Montreal, QC H2X 0A9, Canada; ericbissada@yahoo.com (E.B.); guertinorl@gmail.com (L.G.); 4 Department of Radiation Oncology, Centre Hospitalier de l’Université de Montréal (CHUM), Montreal, QC H2X 0A9, Canada; felixnguyentan@gmail.com (P.F.N.-T.); edith.filion@gmail.com (E.F.); olivier.ballivy.chum@ssss.gouv.qc.ca (O.B.); 5 Department of Medicine, Service of Hemato-Oncology, Centre Hospitalier de l’Université de Montréal (CHUM), Montreal, QC H2X 0A9, Canada; denis.soulieres@umontreal.ca; 6 Department of Radiology, Radio-Oncology and Nuclear Medicine, Université de Montréal, Montreal, QC H3C 3J7, Canada

**Keywords:** autophagy, buparlisib, cancer, combination therapy, chloroquine, HNSCC, omipalisib, oral tongue, PI3K inhibitor, PI3K signaling pathway

## Abstract

Genomic analyses of head and neck squamous cell carcinoma (HNSCC) have highlighted alterations in the phosphatidylinositol 3-kinase (PI3K) signaling pathway, presenting a therapeutic target for multiple ongoing clinical trials with PI3K or PI3K/MTOR inhibitors. However, these inhibitors can potentially increase autophagy in HNSCC and indirectly support cancer cell survival. Here, we sought to understand the relationship between the PI3K signaling pathway and autophagy during their dual inhibition in a panel of HNSCC cell lines. We used acridine orange staining, immunoblotting, and tandem sensor Red Fluorescent Protein- Green Fluorescent Protein-, microtubule-associated protein 1 light chain 3 beta (RFP-GFP-LC3B) expression analysis to show that PI3K inhibitors increase autophagosomes in HNSCC cells, but that chloroquine treatment effectively inhibits the autophagy that is induced by PI3K inhibitors. Using the Bliss independence model, we determined that the combination of chloroquine with PI3K inhibitors works in synergy to decrease cancer cell proliferation, independent of the *PIK3CA* status of the cell line. Our results indicate that a strategy focusing on autophagy inhibition enhances the efficacy of therapeutics already in clinical trials. Our results suggest a broader application for this combination therapy that can be promptly translated to in vivo studies.

## 1. Introduction

Head and neck squamous cell carcinoma (HNSCC) is the seventh most common solid cancer in the USA and the five-year relative survival rate is 66% [1,2]. Platinum-based chemotherapy and radiotherapy are the most common non-surgical therapies, and the few targeted therapies in current clinical use have variable success rates due to frequent resistance development. The Cancer Genome Atlas (TCGA) Research Network (https://www.cancer.gov/about-nci/organization/ccg/research/structural-genomics/tcga) analyzed 530 tumor samples from HNSCC patients and identified the phosphatidylinositol 3-kinase (PI3K) signaling cascade as the most frequently altered pathway with a gain of function in more than 75% of cases. PI3K alterations included *PIK3CA* activating mutation/amplification, phosphatase and tensin homolog (PTEN) protein down-regulation, and Epidermal Growth Factor Receptor (EGFR) amplification [3,4]. Agents targeting the PI3K pathway in HNSCC are under investigation, such as the pan-PI3K pathway inhibitor Buparlisib/BKM120 (Bup) and the dual inhibitor targeting the catalytic sites of PI3K and Mechanistic target of rapamycin (MTOR), Omipalisib/GSK2126458 (Omi). These PI3K inhibitors (PI3Ki) show anti-proliferative activity and decrease the cellular levels of AKT serine/threonine kinase (AKT) phosphorylation as well as the phosphorylation of its downstream effectors [5,6]. Based on promising results that were obtained from cell and animal studies [7,8,9], these inhibitors have been evaluated in clinical trials for solid tumors, including HNSCC cases, and have shown promising results [10,11,12]. In addition to controlling cell proliferation, PI3K signaling is a master regulator of autophagy, which is increasingly recognized as a treatment escape mechanism for tumor cells [13,14]. Autophagy is a homeostatic process for recycling proteins and organelles in the cell and it involves the formation of specific autophagic vacuoles (autophagosomes (AV)) in the cytoplasm. Internal content, such as proteins aggregates and organelles, are degraded by lysosomal enzymes after the fusion of AV with acidic-content lysosomes, giving rise to low pH autolysosomes (AL) [15]. Several stress conditions (starvation, hypoxia, infection) are known activators of autophagy [15,16]). Autophagy in tumor cells promotes their survival by facilitating adaptation to stress and providing access to nutrients in response to the high energy demand and elevated metabolism of cancer cells. However, it must be stated that targeting autophagy in cancer can have negative impact, since autophagy is also important for anti-tumor immunity [13,14].

In vitro work and animal models for HNSCC show that autophagy supports tumor cell growth [17,18,19,20,21] and therapy resistance [22,23,24]. The principal risk factors for HNSCC, such as tobacco, alcohol, and human papilloma virus (HPV), are inducers of autophagy [25]. Studies of autophagy inhibition in cancer patients are ongoing with the only two autophagy inhibitors approved to date for clinical use: chloroquine (CQ) and hydroxychloroquine (HCQ). These drugs have a long history of medicinal use for malaria and autoimmune diseases and are associated with low toxicity and low costs. Encouraging results have emerged from cancer clinical trials [26,27,28]. However, of the 80 clinical trials using CQ or HCQ in combination with standard or new therapeutics, none specifically target HNSCC [29,30]. The published in vitro results for cancer types other than HNSCC demonstrate that PI3Ki in combination with autophagy inhibition are associated with a decrease in cancer cell proliferation and xenograft tumor growth [30,31]. In this study, we hypothesized that PI3Ki use in HSCCC cell lines is associated with the activation of autophagy and explored the combination of PI3Ki and autophagy inhibition as a therapeutic approach to decrease tumor cell proliferation.

## 2. Results

### 2.1. PI3Ki Induce Autophagy Flux in HNSCC Cell Lines

**Figure 1:**
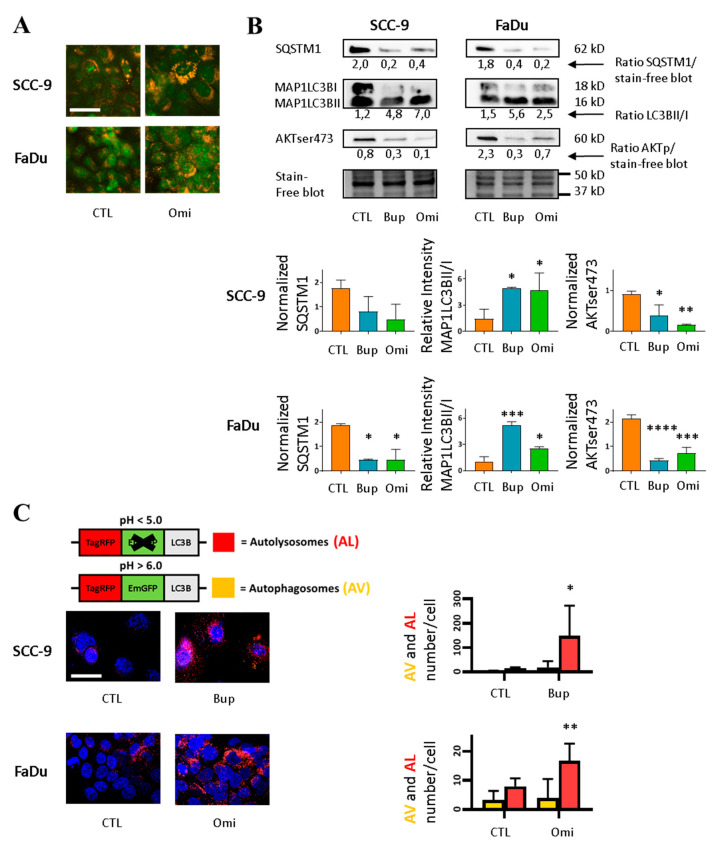
PI3Ki induce autophagy flux in head and neck squamous cell carcinoma (HNSCC) cell lines. (**A**) PI3Ki increase the number of acidic vacuoles. HNSCC cell lines SCC-9 and FaDu were incubated with vehicle dimethyl sulfoxide (DMSO (CTL)) or 1 nM Omi for one day and stained for 15 min. with acridine orange. Nuclei stain green and acidic organelles stain orange. Images are representative of three independent experiments. Scale bar = 50 µm. (**B**) Western blot analysis of autophagy markers and AKTser473 phosphorylation. Top: Autophagy flux is indicated by SQSTM1 degradation and MAP1LC3B conversion, and inhibition of phosphatidylinositol 3-kinase (PI3K) signaling pathway is indicated by decreased AKTser473 phosphorylation. Equal loading of proteins is shown with a stain-free blot. SCC-9 and FaDu were incubated for three days with vehicle, 0.5 µM Bup or 1 nM Omi. Bottom: Bar graphs show densitometric analysis. Data are presented as mean ± SD, *n* = 3 independent experiments and analyzed by ANOVA test. Left: SQSTM1 protein levels relative to stain-free blot or TUBA. For FaDu, * *p* < 0.05 for Bup vs CTL and * *p* < 0.05 for Omi vs CTL. Middle: MAP1LC3B-II protein levels relative to MAP1LC3B-I for SCC-9 (* *p* < 0.05 for Bup vs CTL and Omi vs CTL) and FaDu (*** *p* < 0.001 for Bup vs CTL, * *p* < 0.05 for Omi vs CTL). Right: AKTser473 protein levels relative to stain-free blot or TUBA, for SCC-9: * *p* < 0.05 for Bup vs CTL, ** *p* < 0.01 for Omi vs CTL. For FaDu: **** *p* < 0.0001 for Bup vs CTL, *** *p* < 0.001 for Omi vs CTL. (**C**) Autophagy flux analysis with tandem sensor RFP-GFP-LC3B. PI3Ki increase autophagolysosome (AL) formation. Top: GFP is sensitive to the acidic pH of AL. Red = AL; yellow = autophagosomes (AV). Left: merged stack of confocal images. SCC-9 and FaDu were transduced with the tandem sensor 24 h before the 24 h incubation with inhibitors (1 µM Bup, 5 nM Omi). Cells were stained with 4′,6-Diamidino-2-Phenylindole (DAPI). Scale bar = 50 µm. Right: AL and AV counts per cell of SCC-9 and FaDu using the specialized Image J macro analysis (https://imagejdocu.tudor.lu/plugin/analysis/colocalization_analysis_macro_for_red_and_green_puncta/start). Data are mean ± SD of six images from two independent experiments (three images by experiment per condition with 15–30 cells per picture). For SCC-9 * *p* < 0.05 for Bup vs CTL, and for FaDu ** *p* < 0.01 for Omi vs CTL by unpaired Student’s *t*-test. Uncropped blots are shown in Figure S2.

Human squamous carcinoma cell lines that were derived from oral tongue (SCC-9) and hypopharynx (FaDu) were exposed to PI3Ki to evaluate the functional importance of the PI3K pathway in autophagy in HNSCC. AV fusion to lysosomes was visualized with acridine orange [32], which differentially stains the nuclei in green and the acidic organelles, lysosomes, and AL in orange. Incubation with the PI3Ki Omi increased the number of acidic organelles in HNSCC cell lines, suggesting autophagy induction (Figure 1A).

We used western blot analysis to detect two established markers of autophagy (Figure 1B): microtubule-associated protein 1 light chain 3 beta (MAP1LC3B) and sequestosome 1 (SQSTM1), also known as LC3B and p62, respectively. The cytoplasmic MAP1LC3BI is recruited during AV formation and then cleaved and lipidated to generate MAP1LC3BII. Increased levels of MAP1LC3BII combined with decreased levels of SQSTM1, the autophagosomal cargo protein degraded in the AL, are the hallmarks of autophagic activation [15,16,33]. Consistent with autophagy induction, we observed a decrease in the SQSTM1 levels and an increase in MAP1LC3BII/MAP1LC3BI ratios in the presence of PI3Ki Bup or Omi. We analyzed downstream phosphorylation of AKT at serine 473 to confirm the inhibitor activity towards the PI3K signaling pathway. AKTser473 levels were strongly decreased in HNSCC cell lines by Bup or Omi (Figure 1B).

We also analyzed the autophagic flux with a specific reporter, the Premo^TM^ (Thermo Fisher Scientific, Waltham, MA, USA) autophagy tandem sensor RFP-GFP-LC3B [15,28,34] (Figure 1C top). LC3B targets this fluorescence construct to the AV, and differences in pH sensitivity between Green Fluorescent Protein (GFP) (sensitive to acidic pH) and Red Fluorescent Protein RFP track the progression from AV (green + red LC3B = yellow) to AL (red only). Autophagic flux is assessed by the number and color of the vesicles. The number of red LC3B vesicles increased with PI3Ki Bup or Omi (Figure 1C bottom). Altogether, our results indicate that Bup and Omi are associated with a high autophagic flux and autophagy activation in both cell lines.

### 2.2. PI3Ki and CQ Work in Synergy to Decrease Proliferation of HNSCC Cell Lines

We postulate that the activation of autophagy by PI3Ki enables cancer cells to resist treatment. We performed real-time cell proliferation assays with the IncuCyte S3 Live-Cell Analysis System to verify our hypothesis that the combination of PI3Ki and autophagy inhibitors will lead to decreased tumor cell proliferation (Essen Bioscience, Ann Arbor, MI, USA). The SCC-9 cells were incubated with the autophagy inhibitor CQ or PI3Ki at concentrations that were Federal Drug Administration (FDA) approved for human use or in clinical trial and relevant for patient treatment (as evaluated from blood, plasma and bronchoalveolar fluid samples). More precisely, Bup concentrations in the plasma of cancer patients treated with 100 mg/day were evaluated at 1 μg/mL (2.5 µM) [35]. The ratio of plasma/tumor concentration was evaluated as 1 in a glioblastoma study [36]. Omi at 2 mg/day reaches concentrations of 85 ng/mL in blood (170 nM) and 236 pg/mL (0.5 nM) in bronchoalveolar fluid [37]. Autophagy inhibitor concentrations evaluated in cancer patients from clinical trials were 5 µM in the blood and 1 µM for the plasma [38]. CQ inhibits autophagy by raising the lysosomal pH, which leads to inhibition of both fusion of AV with lysosome and lysosomal protein degradation. As a lysosomotropic agent, CQ preferentially accumulates in the lysosome of the cell and can accumulate in tissues to toxicity levels observed with long-term high dosages used for malaria treatment such that CQ has been associated with retinopathy [30]. Although CQ has several dose-dependent effects, cancer patients could benefited from CQ treatment at a dose of 31 µM (10 mg/kg CQ-Base or 16.7 mg/kg CQ-diphosphate, daily) [39]. We used low concentrations for each inhibitor to measure any enhanced decrease in cell proliferation when inhibitors were used in combination. For CQ, we used a range of concentrations, from 1 to 30 µM.

**Figure 2:**
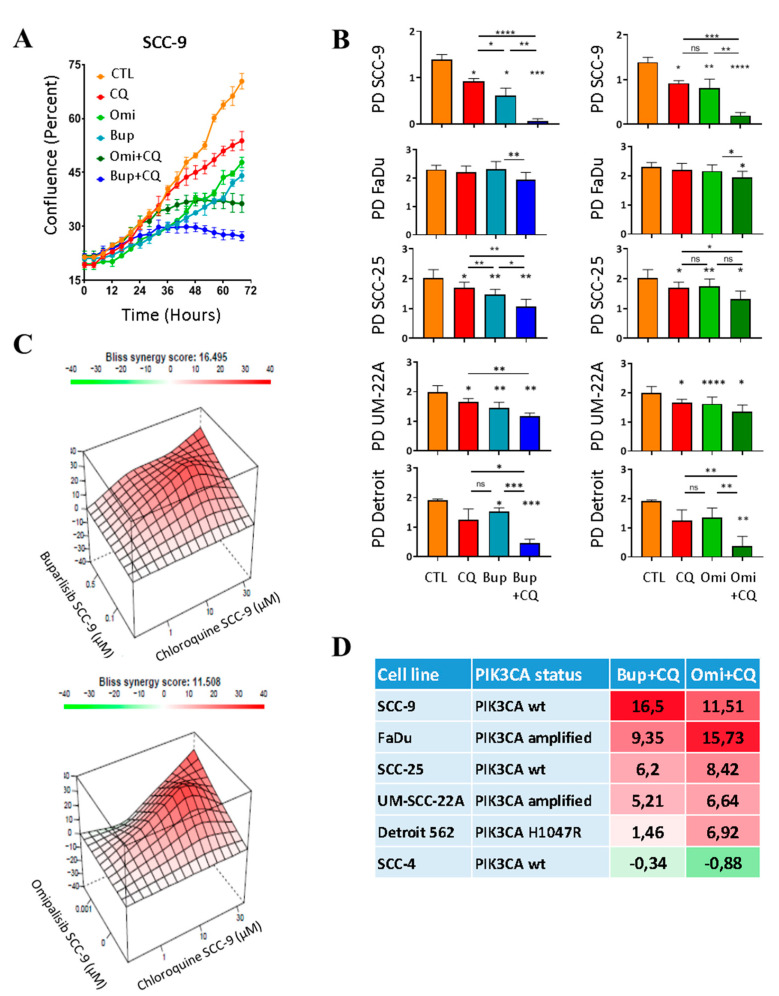
PI3Ki and chloroquine (CQ) work in synergy to decrease proliferation of HNSCC cell lines. (**A**) Real-time cell proliferation of SCC-9 captured by IncuCyte S3 (Essen Bioscience, Ann Arbor, MI, USA) for 3 days with vehicle (CTL), 0.5 µM Bup or 1 nM Omi and ± 10 µM CQ. Data are the mean of triplicates ± SEM. and are representative of four independent experiments. (**B**) Evaluation of population doubling after 3 days in culture for five HNSCC cell lines with/without PI3Ki and CQ at 0.5 µM Bup, 1 nM Omi and 10 µM CQ (except for Detroit 562: 1 µM Bup, 10 nM Omi, and 30 µM CQ). Data are the mean ± SD; *n* = 4 independent experiments in triplicates for SCC-9, UM22A, and Detroit 562, and *n* = 5 in triplicates for FaDu and SCC-25. Data were analyzed with ANOVA test, * *p* < 0.05, ** *p* < 0.01, *** *p* < 0.001, and **** *p* < 0.0001. * *p* without underscore bar represents significant difference compared with CTL (vehicle). (**C**) Three-dimensional (3D) diagrams representing Bliss score (z) versus PI3Ki concentration (y) and autophagy inhibitor concentration (x). SCC-9 cell line was incubated with different concentrations of the inhibitors alone or in combination (1, 10, 30 µM CQ; 0.1 or 0.5 µM Bup; 0.1 or 1 nM Omi). Real-time proliferation was recorded for four days with IncuCyte S3 (Essen Bioscience, Ann Arbor, MI, USA) before AUC determination and Bliss score evaluation. Top: Bup. Bottom: Omi. (**D**) Bliss synergy scores for treatment combinations and *PIK3CA* status of six HNSCC cell lines. Bliss synergy scores over 1 indicate synergy. Cell lines were incubated in IncuCyte S3 (Essen Bioscience, Ann Arbor, MI, USA), as described with different concentrations of the inhibitors alone or in combination (1, 10, 30 µM CQ; 0.1 or 0.5 µM Bup; 0.1 or 1 nM Omi with the exception of FaDu with 1 or 5 nM Omi, and of Detroit 562 with 0.2 or 1 µM Bup and 2 or 10 nM Omi). Real-time proliferation was recorded for 3-4 days with IncuCyte S3 (Essen Bioscience, Ann Arbor, MI, USA).

We observed a decrease in the proliferation of SCC-9 and FaDu cells in response to CQ or PI3Ki treatment (Figure 2A and Figure S1A). The effect of PI3Ki on proliferation was significantly enhanced when CQ was added to the treatment. We performed the same experiment with a panel of HPV-negative HNSCC cell lines harboring the most common *PIK3CA* alterations (Figure 2D), but all mutated for *TP53* [40,41,42,43,44]. *TP53* mutated cell lines represent 86% of HPV-negative HNSCC cases [3], and a study found *TP53* mutations in 95% of metastatic HNSCC [45]. Real-time cell proliferation assays for this panel showed the same phenomenon: population doubling (PD) was significantly decreased with the combination of CQ and PI3Ki in five of six HNSCC cell lines (SCC-4 not shown, Figure 2B). SCC-9 dose-response curves for each inhibitor, alone or in combination, were analyzed while using the SynergyFinder software [46] with the Bliss independence model [47,48] showing high synergy between PI3Ki Bub or Omi and CQ (Figure 2C). Bliss synergy analysis for the other HNSCC cell lines of the panel (Figure S1B) demonstrated a high synergy between PI3Ki and CQ, as summarized in Figure 2D, with similar trends in cell line sensitivity for Bup and Omi and appeared independent of *PIK3CA* mutation/amplification status. Our results advocate for the use of a combination therapy in HNSCC, which would include CQ in addition to PI3Ki.

### 2.3. CQ Inhibits PI3Ki-Induced Autophagy

**Figure 3:**
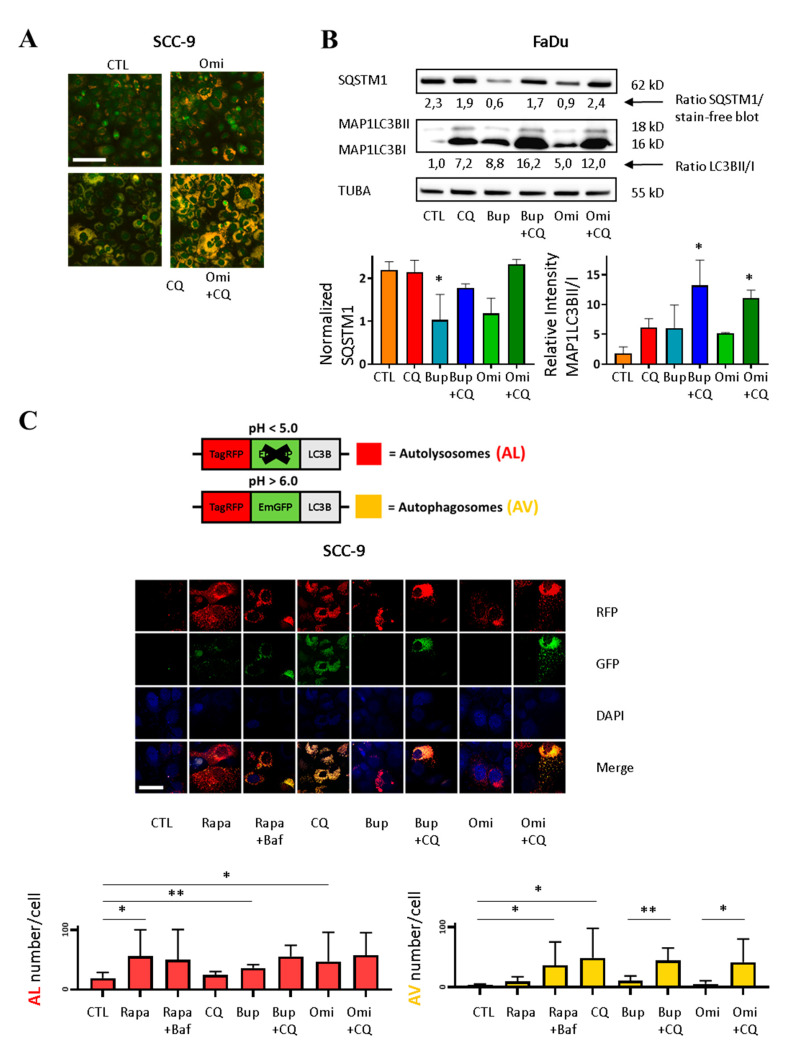
CQ inhibits PI3Ki-induced autophagy in HNSCC cell lines. (**A**) SCC-9 cells were incubated with vehicle DMSO (CTL), 1 nM Omi, 10 µM CQ or both inhibitors for one day and stained for 15 min. with acridine orange. Nuclei stain green and acidic organelles stain orange. Images are representative of three independent experiments. Scale bar = 100 µm. (**B**) Top: Western blot of autophagy markers SQSTM1 and MAP1LC3B, using alpha-tubuline as a loading control. FaDu cells were incubated for 3 days with vehicle, 0.5 µM Bup or 1 nM Omi and ± 10 µM CQ. Bottom: Densitometric analysis of SQSTM1 protein levels relative to TUBA and of MAP1LC3B-II protein levels relative to MAP1LC3B-I. Data are presented as mean ± SD, *n* = 3 independent experiments and analyzed by ANOVA test. * *p* < 0.05 for normalized SQSTM1 Bup vs CTL and for relative MAP1LC3B Bup + CQ vs CTL and Omi + CQ vs CTL. (**C**) Autophagy flux analysis with tandem sensor RFP-GFP-LC3B. Top: GFP is sensitive to acidic pH of AL. Red = AL, yellow = AV. Bottom: Confocal images and AL and AV counts per cell with specialized Image J macro analysis. SCC-9 cells were transduced with the tandem sensor 24 h before 24 h incubation with inhibitors: 1 nM Rapa, 10 µM CQ. Baf (0.3 uM) was added for the last 4 h. The cells were stained with DAPI. PI3Ki concentrations were 0.5 µM Bup and 1 nM Omi. Scale bar = 50 µm. Data are presented as mean ± SD, six images from two independent experiments (three images by experiment per condition with 15–30 cells per picture) and analyzed by unpaired Student’s t-test: * *p* < 0.05 for AL Rapa and AL Omi vs AL CTL and ** *p* < 0.01 for AL Bup vs AL CTL, * *p* < 0.05 for AV Rapa + Baf and AV CQ vs AV CTL and between AV Omi and AV Omi + CQ, ** *p* < 0.01 for AV Bup vs AV Bup + C. Uncropped blots are shown in Figure S2.

Our results suggest that the induction of autophagy associated with the concomitant inactivation of the PI3K pathway is blocked by CQ. To verify this, SCC-9 cells were incubated for one day with Omi, CQ, or the combination of both and stained with acridine orange. Incubation with PI3Ki Omi increased the number of acidic organelles in the HNSCC cell lines due to autophagy activation (Figure 3A). We observed an increase in acidic organelle content with CQ, which inhibits autophagy at a late stage: the fusion of AV to lysosomes is blocked leading to AV accumulation. We observed a further increase of acidic organelles with the combination, suggesting the accumulation of acidic organelles caused by an inhibition of PI3Ki-induced autophagy. We made the same observation when FaDu cells were exposed for one day to Bup, CQ, or both inhibitors (Figure S1C). We examined two markers of autophagy previously described to analyze the role of each pathway in the combination setting: MAP1LC3BII and SQSTM1. We observed a decrease of SQSTM1 and an increase of MAP1LC3BII levels in the presence of PI3Ki. With CQ, SQSTM1 degradation was prevented and MAP1LC3BII accumulated in the cells. The MAP1LC3BII levels were further increased by the combination treatments, showing that CQ blocked autophagy induced by PI3K inhibition (Figure 3B). We also analyzed the autophagic flux with the tandem RFP-GFP-LC3B sensor (Figure 3C top). AVs have been shown to accumulate following the addition of drugs, such as CQ and the autophagy inhibitor Bafilomycine A1 (Baf), which blocks autophagy through lysosomal pH neutralization and prevents fusion with lysosomes [15,28,34]. The number of red LC3B vesicles increased with rapamycin (Rapa), an MTOR inhibitor that is known to induce autophagy [15], and with PI3Ki, Bup, or Omi, as shown previously. We observed AV (yellow vesicles) accumulation with Rapa + Baf incubation as well as with CQ. AV number by cell was also significantly increased by treatment combinations of CQ and PI3Ki (Figure 3C bottom), demonstrating that CQ inhibits PI3Ki-induced autophagy.

## 3. Discussion

In this study, we demonstrated that autophagy was activated by two PI3Ki with different mechanisms of action: a pan-class I PI3K family lipid kinase inhibitor, Bup, and a dual PI3K/MTOR inhibitor, Omi. In both cases, CQ prevented the PI3Ki-induced autophagy, as indicated by AV accumulation. We propose that autophagy in HNSCC tumor cells is protective, as tumor cells use autophagy for ROS protection and as an energy source [13,14]. In this context, CQ blocks a tumor escape mechanism to treatment and provides a therapeutic approach that combines CQ with PI3Ki for a better anti-proliferative effect.

We used *TP53* mutated HPV-negative HNSCC cell lines, which was a limitation in representing the range in heterogeneity of HNSCC found by the TCGA study [3,4]. HPV-positive HNSCC was not evaluated, as it is a very different disease, mostly relevant for oropharyngeal cancer and responds much better to current therapies [49]. Current clinical studies for HPV-positive HNSCC mostly investigate a potential reduction of therapy instead of adding new treatments for patients who usually have a good prognosis. On the other hand, HPV-negative HNSCC therapy is in need of improvement. *TP53* status has been shown to influence responses to autophagy inhibition. For example, in *TP53* null mice, autophagy inhibition was shown to promote cancer growth [50]. In this context, we focused on *TP53* mutated HNSCC which is most frequent among HPV-negative HNSCC. Moreover, a recent computational analysis of the *TP53* mutational landscape in HNSCC showed that specific mutations were associated with distinct pathobiological pathways and prognostic signatures [51].

CQ or HCQ have shown limited efficacy as monotherapies in clinical trials. The same is true for PI3K signaling pathway inhibitors. Our results show that the efficacy of combination treatment was not dependent on *PIK3CA* tumor status. Early studies showed that Bup exhibited preferential inhibition of tumor cells bearing *PIK3CA* mutations, in contrast to either *KRAS* or *PTEN* mutant models [5]. In patients, *PIK3CA* mutations were neither necessary nor predictive of the response to Omi or Bup treatment (Bup in combination with paclitaxel) [10,11]. Our results are in line with these observations and they indicate that other elements are involved in the cell response to treatment. Recent studies in mutant KRAS- or BRAF-driven mouse models of cancer, such as pancreatic ductal adenocarcinoma cancer (PDAC), demonstrated synergistic antitumor activity with dual autophagy inhibition and mitogen-activated protein kinase kinase (MEK) or mitogen-activated protein kinase (ERK) inhibitors [28,34]. The RAF-MEK-ERK signaling pathway is another major signaling pathway that controls cell proliferation. These results, along with our findings and other studies focusing on PI3K pathway inhibition, suggest that therapeutic strategies using inhibitors of a major cell proliferation signaling pathway, such as PI3K or MAPK, disrupt the signaling cascade and drive cells toward autophagy, enhancing cell responsiveness to autophagy inhibition. This strategy must be adapted to the type of cancer (PI3Ki for HNSCC or MAPK inhibitors for PDAC), and also to the genetic variants/subtypes within each cancer. For example, the TCGA analysis of HNSCC suggests that a small proportion of cases are a MAPK-dependent type of cancer [3]. We anticipate that the development of precision medicine will help to determine the leading proliferative pathway in each individual cancer, allowing for better therapeutic choices for improved treatment outcomes. This will be possible with tumor genetic sequencing for oncogene mutations, but also with ex-vivo testing of inhibitors on tumor fragments before chemotherapy treatment [52]. Investigation into the long-term inhibitory effects of these pathways in normal or cancer cells are still at an early stage and require further study for their safe use in personalized therapies.

## 4. Materials and Methods

### 4.1. Cell Culture

Human squamous carcinoma cell lines of the oral tongue (SCC-4, SCC-9, SCC-25), hypopharynx (FaDu), and of a metastasis from the pharynx (Detroit 562) were purchased from the American Type Culture Collection (Manassas, VA, USA) (CRL-1624, CRL-1629, CRL-1628, HTB-43, and CCL-138). The UM-SCC-22A (hypopharynx) cell line was purchased from EMD Millipore Corporation (Burlington, MA, USA) (SCC076). The cells were grown in Dulbecco’s Modified Eagle’s Medium (DMEM) (Gibco™, 11995-065; Waltham, MA, USA) supplemented with 10% fetal bovine serum (Wisent, 088150; Saint-Jean-Baptiste, QU, Canada), 1% penicillin/streptomycin (Wisent, 450-201-EL; Saint-Jean-Baptiste, QU, Canada), and 1 times non-essential amino acids (Gibco™, 11140-050; Waltham, MA, USA), and they were used before the tenth passage from the first thaw after reception. All of the cell lines are HPV-negative.

### 4.2. IncuCyte^®^ Live-Cell Analysis for Cell Proliferation and Bliss Synergy Score

Cells were seeded in 96-well plates, 100 µL per well, and allowed to adhere overnight. SCC-4, SCC-9, and SCC-25 were seeded at a density of 3500 cells/well; FaDu and UM-SCC-22A at 5000 cells/well; and, Detroit 562 at 10,000 cells/well. The following day, 100 µL of media containing 2 times concentration of vehicle or inhibitor or combination of inhibitors was added in triplicates. PI3Ki stock solutions were prepared in 100% dimethyl sulfoxide (DMSO). Working solutions were prepared fresh before addition to the cell media, such that final DMSO concentrations were kept constant in both control and treated cells. Proliferation assays consisted of longitudinal imaging of the cell layer and confluence evaluation with the IncuCyte S3 system (Essen Bioscience, Ann Arbor, MI, USA). Phase contrast images of the full well were acquired at 4-h intervals for 72 h at 4 times magnification. The proliferation curves were generated using the confluence evaluation algorithm of the IncuCyte S3 software. PD was calculated using this formula: log2 (confluence at 72 h/confluence at 0 h).

### 4.3. Bliss Synergy Score Calculation

The cells were incubated, as described above, with vehicle DMSO or different concentrations of the inhibitors, alone or in combination. Real-time proliferation was recorded for 3–4 days at 4-h intervals with IncuCyte S3 (Essen Bioscience, Ann Arbor, MI, USA). Area under the curve (AUC) with baseline correction was calculated using GraphPad version 8 (https://www.graphpad.com) when control conditions reached 75–80% confluence, as evaluated with the IncuCyte S3 software. AUC results were expressed as % proliferation versus control and formatted in a matrix for analysis with SynergyFinder software [46]. A Bliss synergy score over 1 indicates synergy.

### 4.4. Acridine Orange Staining

Cells were seeded in 96-well plates, 100 µL per well, and allowed to adhere overnight. The following day, 100 µL of media containing 2 times the concentration of vehicle or inhibitor or inhibitor combinations was added in triplicates. The cells were incubated for 24 h and acridine orange was added at a final concentration of 5 µg/mL for 15 min. Images of phase contrast, and green and red fluorescence were acquired with the IncuCyte S3 system (Essen Bioscience, Ann Arbor, MI, USA) at 20 times magnification of two images per well.

### 4.5. Immunoblotting

Cellular proteins were extracted, separated by electrophoresis, transferred to nitrocellulose membranes, and probed, as described previously [16]. The antibodies used for western blotting were anti-LC3B (Novus, NB600-1384, Saint Charles, MI, USA; Cell Signaling Technology, 12741S, Danvers, MA, USA), anti-human SQSTM1 (Abcam, ab56416; Cambridge, MA, USA), and anti-AKTser473 (Cell Signaling Technology, 9271; Danvers, MA, USA). Membranes were stained with Ponceau S Red (Sigma, P-3504; ST-Louis, MO, USA) for protein visualization. After initial probing, membranes were stripped with Restore Plus western blot stripping buffer (Thermo Fisher Scientific, 46430; Waltham, MA, USA) and then re-probed with anti-alpha-tubulin as a loading control (Cell Signaling Technology, 3873; Danvers, MA, USA). Alternatively, cellular proteins were separated by electrophoresis in stain-free gels (Bio-Rad, 456-8124; Hercules, CA, USA) for direct visualization and quantification of the proteins. Densitometric analyses were conducted with the ImageLab software from BioRad (version 1.0; Hercules, CA, USA). Data are expressed in arbitrary units.

### 4.6. Tandem Sensor RFP-GFP-LC3B Expression

We used the Premo^TM^ autophagy tandem sensor RFP-GFP-LC3B BacMam 2.0 expression vector kit from Thermo Fisher (P36239; Waltham, MA, USA) and followed instructions that were provided by the manufacturer. In brief, the HNSCC cell lines were plated in 24-well plates containing coverslips at a density of 30,000 cells/well for SCC-9 and 40,000 cells/well for FaDu. The day after, the cells were infected with baculovirus at a multiplicity of infection of 30 in complete medium. After overnight incubation, cells were exposed to experimental conditions for 24 h unless otherwise stated. Cells were then fixed with formalin for 15 min., washed with PBS, and coverslips were mounted onto slides with Prolong Gold^®^ anti-fade reagent with DAPI (Thermo Fisher, P36935; Waltham, MA, USA). Cells were observed using a confocal microscope (Leica TCS SP5 MP; Concord, ON, Canada). Three pictures (64 times magnification), each capturing approximately 15–30 cells, were taken per condition. The Image J software with a macro adapted from Daniel J. Shiwarski, Ruben K. Dagda and Charleen T. Chu (https://imagejdocu.tudor.lu/plugin/analysis/colocalization_analysis_macro_for_red_and_green_puncta/start) was used for quantification.

### 4.7. Reagents

Buparlisib (NVP-BKM120) (11587) was purchased from Cayman Chemical, Ann Arbor, MI, USA; Omipalisib (GSK2126458) (S2658) from Selleckchem, Houston, TX, USA; Rapamycin (R0161) from LKT Labs, St. Paul, MN, USA; Bafilomycine A1 (B1793), Chloroquine (C6628), and phosphatase inhibitor cocktail 2 (5726) from Sigma, ST-Louis, MO, USA; protease inhibitor cocktail 3 (535140) from Calbiochem, San Diego, CA, USA; and acridine orange (A-1301) from Thermo Fisher, Waltham, MA, USA. All other reagents were from Sigma Chemicals, ST-Louis, MO, USA.

### 4.8. Statistical Analysis

Comparisons of PD among different treatment groups were made using Kruskal–Wallis one-way analysis of variance (ANOVA) for matched data with post-hoc Tukey HSD test with the GraphPad Prism software version 8 (https://www.graphpad.com/scientific-software/prism). Bar graphs were presented as mean ± standard deviation (SD). Values of * *p* < 0.05, ** *p* < 0.01, *** *p* < 0.001, **** *p* < 0.0001 were considered to be significant. * *p* without underscore bar represents a significant difference with the control (DMSO vehicle). Other results, expressed as mean ± SD, were analyzed by unpaired Student’s t-test. Values of * *p* < 0.05, ** *p* < 0.01, *** *p* < 0.001, **** *p* < 0.0001 were considered significant. * *p* without underscore bar represents a significant difference with the control (DMSO vehicle).

## 5. Conclusions

We showed that HNSCC cell lines that were exposed to PI3Ki displayed increased autophagy and that autophagy inhibitor CQ blocked the PI3Ki-induced autophagy flux. PI3Ki used in conjunction with CQ demonstrated a synergism that enhanced the inhibition of HNSCC cell proliferation and was independent of the *PIK3CA* status of the cell lines. We present a potential therapeutic strategy of adding CQ to the PI3Ki treatment in HNSCC that is not dependent on the *PIK3CA* status of the tumor. These results can be translated to in vivo studies and have implications for the design of future clinical trials.

## Supplementary Materials

The following are available online at https://www.mdpi.com/2072-6694/12/9/2371/s1, Figure S1: PI3K inhibitors and autophagy, Figure S2: Uncropped Western blots.
