# Supplementary material for: Dual Inhibition of Autophagy and PI3K/AKT/MTOR Pathway as a Therapeutic Strategy in Head and Neck Squamous Cell Carcinoma
Source: Cancers (Basel). 2020 Aug 21;12(9):2371. doi: 10.3390/cancers12092371 (PMC7563873; doi:10.3390/cancers12092371)
Supplement: Supplementary file 1 [file cancers-12-02371-s001.pdf]

Supplementary Material: Dual Inhibition of Autophagy and PI3K/AKT/MTOR Pathway as a Therapeutic Strategy in Head and Neck Squamous Cell Carcinoma

Monique Bernard, Guillaume B. Cardin, Maxime Cahuzac, Tareck Ayad, Eric Bissada, Louis Guertin, Houda Bahig, Phuc Felix Nguyen-Tan, Edith Filion, Olivier Ballivy, Denis Soulieres, Francis Rodier and Apostolos Christopoulos

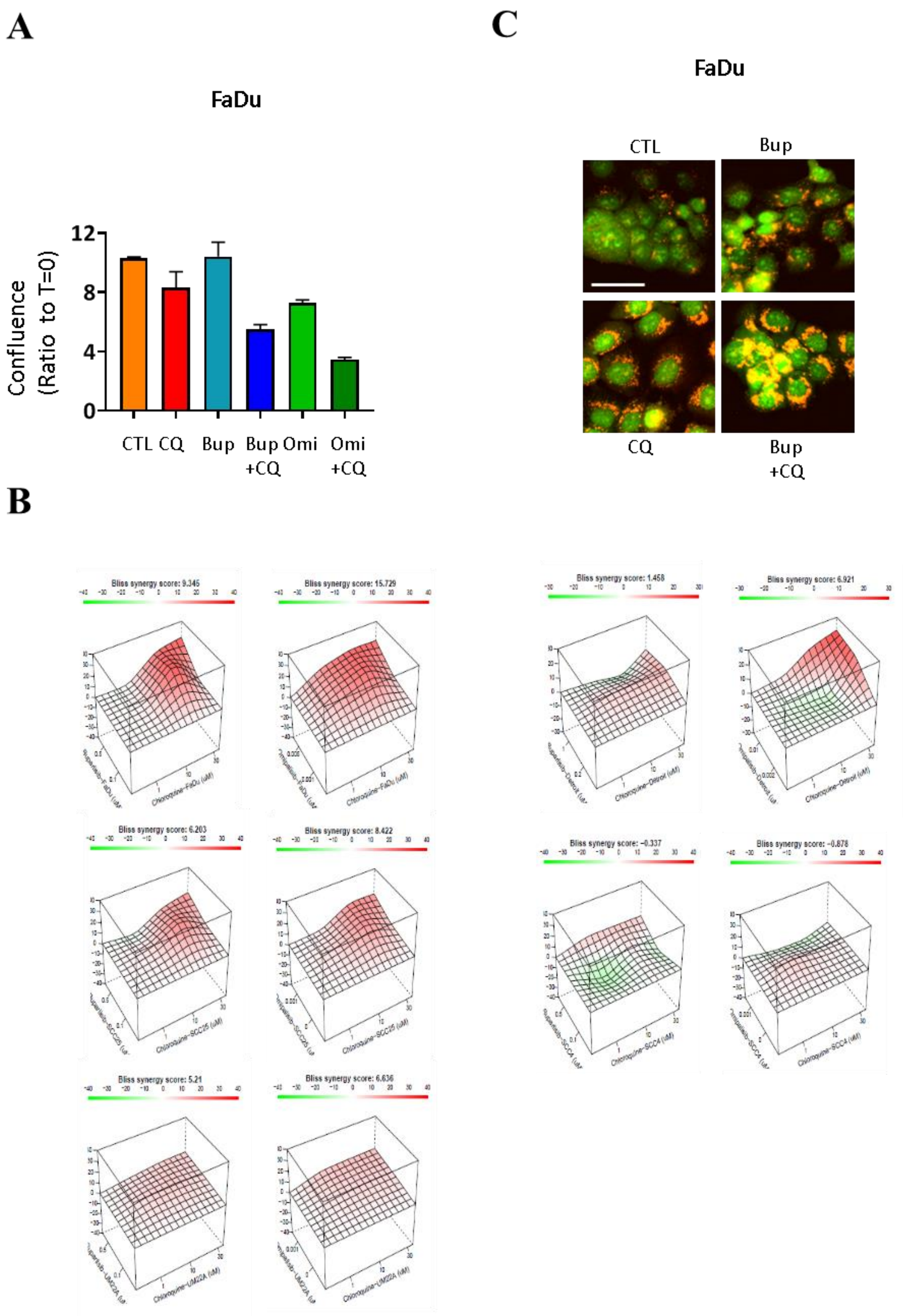

Figure 1. PI3K inhibitors and autophagy. (**A**) Real time cell proliferation of FaDu. Cells were incubated for 4 days with vehicle (CTL), 0.5 µM Bup or 5 nM Omi and ± 10 µM CQ, confluence was captured and evaluated with IncuCyte S3. Data are the mean of triplicates ± SEM of confluence at the end of the experiment normalized to confluence at time 0 and are representative of 3 independent experiments. (**B**) Bliss score 3D diagrams. Three-dimensional (3D) diagrams representing Bliss score (z) versus PI3Ki concentration (y) and autophagy inhibitor concentration (x). HNSCC cell lines were incubated with different concentrations of the inhibitors alone or in combination (1, 10, 30 µM CQ; 0.1 or 0.5 µM Bup; 0.1 or 1 nM Omi with the exception of FaDu with 1 or 5 nM Omi, and Detroit 562 with 0.2 or 1 µM Bup and 2 or 10 nM Omi). Real-time proliferation was recorded for up to 4 days with IncuCyte S3 before AUC determination and Bliss score evaluation. (**C**) Acridine orange staining of FaDu cell line with Bup. FaDu cells were incubated with vehicle DMSO (CTL), 1 µM Bup, 10 µM CQ or both inhibitors for 1 day and stained for 15 min with acridine orange. Images are representative of 3 independent experiments. Scale bar = 50 µm.

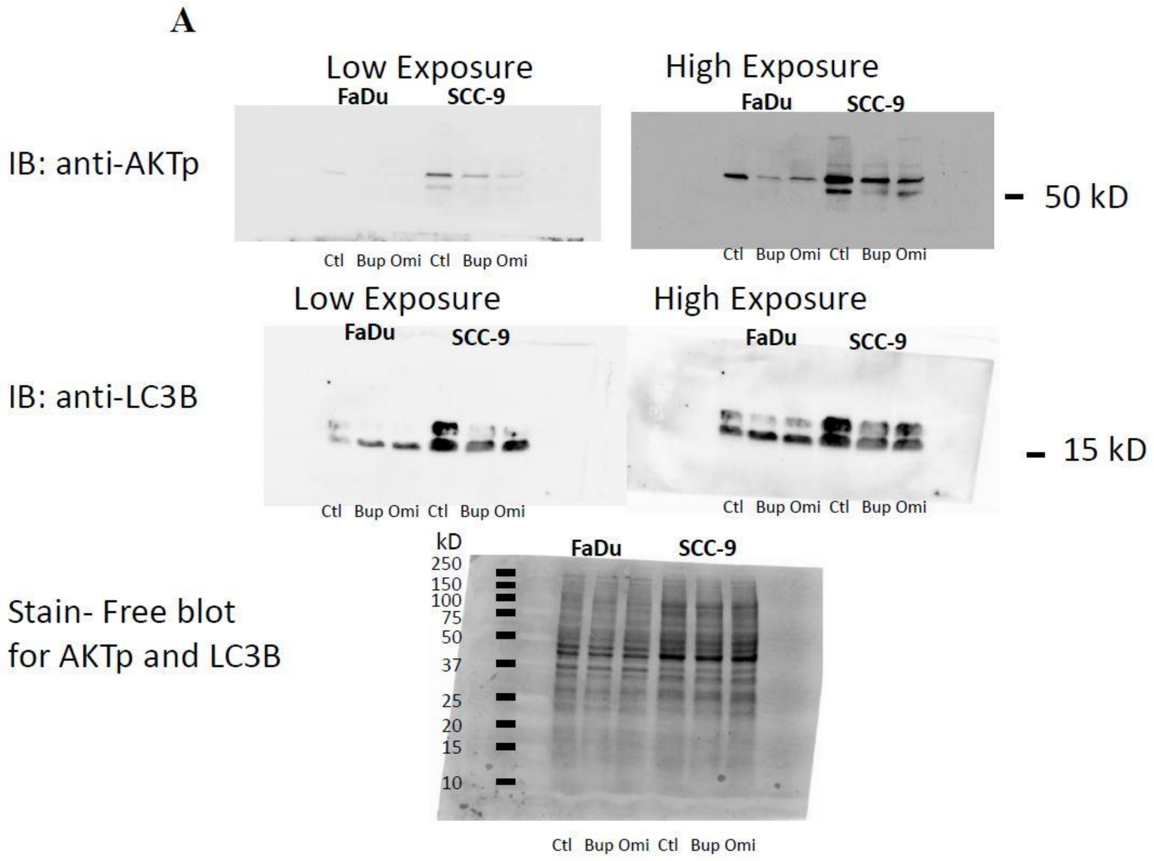

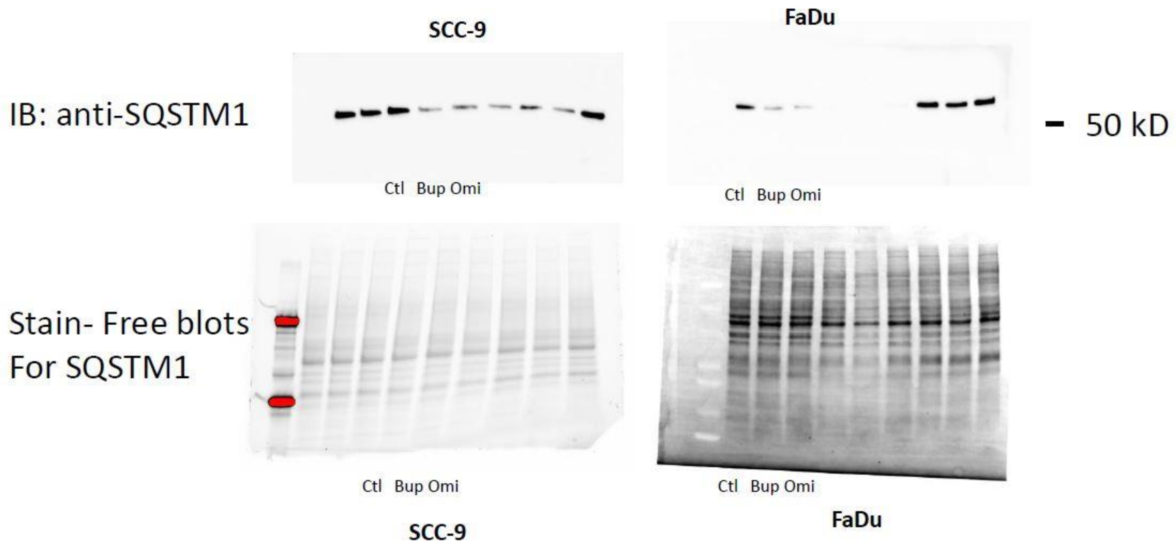

B

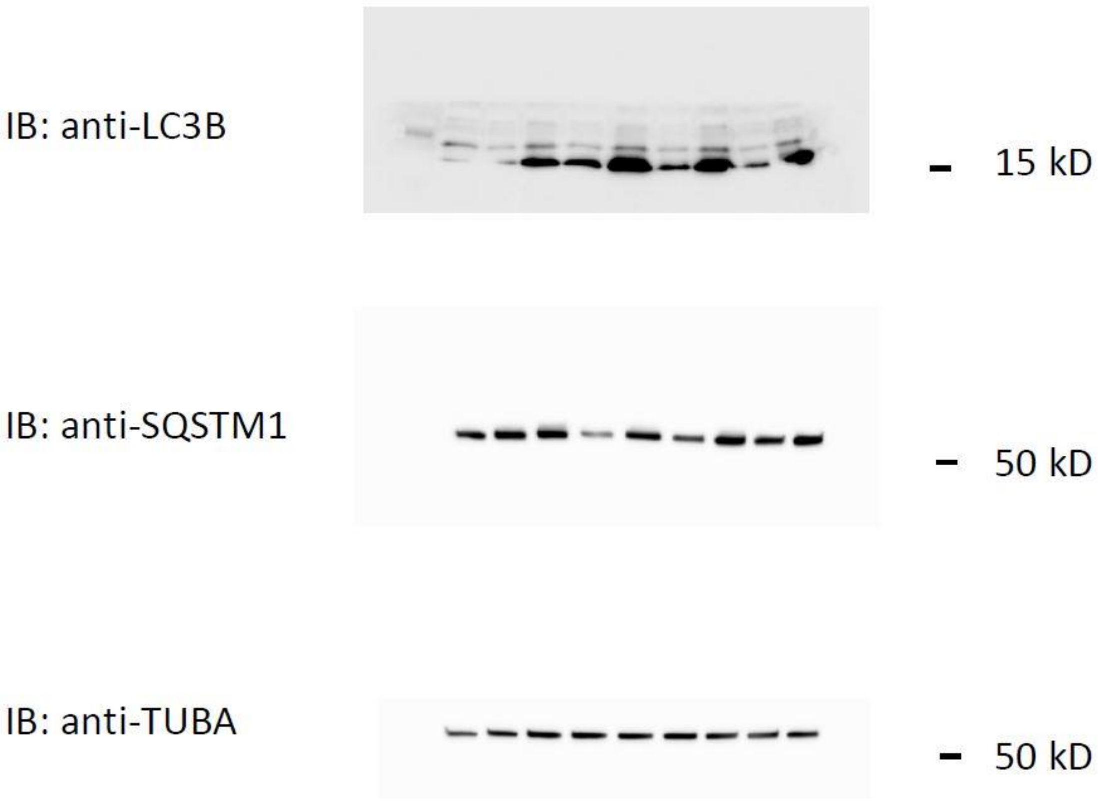

Figure 2. Uncropped Western blots. (**A**) The uncropped SDS-PAGE images of figure 1B. (**B**) The uncropped SDS-PAGE images of figure 3B.

© 2020 by the authors. Licensee MDPI, Basel, Switzerland. This article is an open access article distributed under the terms and conditions of the Creative Commons Attribution (CC BY) license (http://creativecommons.org/licenses/by/4.0/).
